# Extrapolation of praziquantel pharmacokinetics to a pediatric population: a cautionary tale

**DOI:** 10.1007/s10928-018-9601-1

**Published:** 2018-09-14

**Authors:** Peter L. Bonate, Tianli Wang, Paul Passier, Wilhelmina Bagchus, Howard Burt, Christian Lüpfert, Nada Abla, Jana Kovac, Jennifer Keiser

**Affiliations:** 1 Astellas, 1 Astellas Way, Northbrook, IL 60062 USA; 2 Present Address: Alkermes, Waltham, MA 02451 USA; 3 Present Address: Galapagos BV, Zernikedreef 16, Leiden, The Netherlands; 4 Merck Serono SA, Merck Institute for Pharmacometrics (A Subsidiary of Merck KGaA, Darmstadt, Germany), Lausanne, Switzerland; 5 Simcyp (a Certara company), Blades Enterprise Centre, John Street, Sheffield, S2 4SU UK; 6 Merck KGaA, Translational Quantitative Pharmacology, Frankfurter Str. 250, 64293 Darmstadt, Germany; 7 Merck Global Health Institute, Ares Trading S.A. (A Subsidiary of Merck KGaA, Darmstadt, Germany), 1262 Eysins, Switzerland; 8 Swiss Tropical and Public Health Institute, Socinstr. 57, CH-4002 Basel, Switzerland; 9 University of Basel, Basel, Switzerland

**Keywords:** Population pharmacokinetics, NONMEM, Linear mixed effects models, Root cause analysis, Oral dispersion tablet, Complex pharmacokinetics

## Abstract

L-praziquantel (PZQ) pharmacokinetic data were analyzed from two relative bioavailability Phase 1 studies in adult, healthy subjects with two new oral dispersion tablet (ODT) formulations of L-PZQ administered under various combinations of co-administration with food, water, and/or crushing. Linear mixed effects models adequately characterized the noncompartmental estimates of the pharmacokinetic profiles in both studies. Dose, food, and formulation were found to significantly affect L-PZQ exposure in both studies. The model for AUC was then extrapolated to children 2–5 years old accounting for enzyme maturation and weight. The predicted exposures were compared to an external Phase 1 study conducted by the Swiss Tropical and Public Health Institute using a currently marketed formulation (Cesol 600 mg immediate-release tablets) and found to be substantially lower than observed. A root cause analysis was completed to identify the reason for failure of the models. Various scenarios were proposed and tested. Two possible reasons for the failure were identified. One reason was that the model did not account for the reduced hepatic clearance seen in patients compared to the healthy volunteer population used to build the model. The second possible reason was that PZQ absorption appears sensitive to meal composition and the model did not account for differences in meals between a standardized Phase 1 unit and clinical sites in Africa. Further studies are needed to confirm our hypotheses.

**Electronic supplementary material:**

The online version of this article (10.1007/s10928-018-9601-1) contains supplementary material, which is available to authorized users.

## Introduction

Praziquantel (PZQ) is the current gold standard treatment for schistosomiasis, one of the most neglected tropical diseases that remains one of the most prevalent parasitic diseases in developing countries. Treatment and control of schistosomiasis is caused primarily by three main schistosome species, *Schistosoma haematobium*, *S. japonicum* and *S. mansoni*, and relies exclusively on PZQ [1]. PZQ was co-developed by Bayer and Merck in the 1970s and commercialized under the name of Biltricide^®^ 600 mg (Bayer), Cisticid^®^ 600 mg and Cisticid^®^ 500 mg (Merck KGaA) for human use. Other generic PZQ products are also marketed worldwide. Both products exist as a 1:1 racemic mixture with L-PZQ (or R-(−)-Praziquantel) being the biologically active enantiomer and the D-isomer (or (S-(+)-Praziquantel) being the inactive enantiomer mostly responsible for its bitter taste [2–4]. The absorption of PZQ from the gastrointestinal tract is nearly complete, with a peak concentration reached within 1–2 h. Due to extensive first-pass metabolism, as little of the drug is excreted unchanged, almost exclusively via the renal path, PZQ has a short half-life of 1–3 h in both healthy normal volunteers and infected adults [4].

The prevalence of schistosomiasis among Sub-Saharan children is very high. In 2015, 53.2 million of 118 million school-age children in need for treatment received preventive chemotherapy for schistosomiasis [5]. Current treatment is a single dose of 40 mg/kg using 500 mg or 600 mg PZQ tablets. The large size of the commercially available PZQ tablets makes it difficult, especially for young children, to swallow. Hence, PZQ in this population is mostly administered after crushing the tablet. While school-age children are recognized as one of the most affected populations and regularly treated, pre-school children were until recently not considered. Nonetheless, it was shown that schistosomiasis among young children is very common and there is consensus that they should be included in treatment programs [6]. To address the gap of non-treatment of pre-school age children, the Pediatric Praziquantel Consortium (http://www.pediatricpraziquantelconsortium.org) was established under the umbrella of Lygature (Utrecht, the Netherlands) with partners from the pharmaceutical industry (Merck KGaA, Germany, Astellas Pharma, Japan and SimCyp, United Kingdom), the academic sector (Swiss Tropical and Public Health Institute, Swiss TPH), as well as Fiocruz foundation attached to the Brazilian Ministry of Health. The Schistosomiasis Control Initiative (SCI), part of Imperial College London, joined the Consortium in 2016. The consortium aspires to develop a new pediatric formulation of PZQ and register its use in the pediatric schistosomiasis indication. In the framework of the development of the pediatric formulation, two Phase 1 pharmacokinetic studies were conducted in healthy adult volunteers. The objectives of this current analysis were to characterize L-PZQ pharmacokinetics in adult, healthy subjects enrolled in these Phase 1 studies and to extrapolate these results to those obtained from a Phase 2 study in an African pediatric population infected with *S. mansoni* in order to determine the equivalent pediatric dose for use in a Phase 2 study in the target populations of children 2–6 years old to be conducted under the auspices of the PZQ Pediatric Consortium development program.

## Methods

### Overview of studies 200585-001 and 200661-001

Study 200585-001 (https://clinicaltrials.gov/ct2/show/NCT02325713?term=200585-001&rank=1) was a Phase 1, open-label, randomized, four-period, crossover, single center trial to assess the relative bioavailability of a single oral dose of the new 150 mg Oral Dispersible Tablet (ODT) formulation of PZQ at different dose levels versus the current commercial 500 mg tablet formulation of PZQ in healthy male subjects. The primary objective of the trial was to assess the relative bioavailability of the newly developed racemic ODT-PZQ tablet of 150 mg dispersed in water versus the current racemate Cysticide^®^ tablet of 500 mg after single oral administration at a dose of 40 mg/kg in healthy subjects under fed conditions.

Subjects were dosed in a 4-period crossover in different cohorts for logistic reasons with a 7 day washout between each administration of study drug. Treatments were:

(A) Racemic ODT-PZQ formulation at 40 mg/kg dispersed in water after a meal uncrushed (Period 1 and 2) (n = 30).
(B) Current PZQ formulation (Cysticide) at 40 mg/kg given with water after a meal uncrushed (Period 1 and 2) (n = 30).
(C) Racemic ODT-PZQ formulation at 20 mg/kg dispersed in water after a meal (C1) or at 60 mg/kg dispersed in water after a meal (C2) uncrushed (Period 3 and 4) (C1 n = 14, C2 n = 15).
(D) Racemic ODT-PZQ formulation at 40 mg/kg dispersed in water without a meal (D1) or current PZQ formulation at 40 mg/kg given as crushed tablets (using a mortar and pestle) with water after a meal (D2) (Period 3 and 4) (D1 n = 14, D2 n = 14).

Study 200661-001 (https://clinicaltrials.gov/ct2/show/NCT02271984?term=200661-001&rank=1) was a Phase 1, open-label, randomized, single dose, five period, crossover, single center trial to assess the relative bioavailability of the 150 mg ODT formulation of L-PZQ vs. the current 500 mg PZQ commercial racemate tablet formulation in healthy male subjects. The ODT formulation in Study 200661-001 was different than Study 200585-001 in that Study 200661-001 was a pure enantiomeric ODT while Study 200585-001 was a racemic ODT. The primary objective of the trial was to assess the relative bioavailability of the recently developed L-PZQ 150 mg ODT tablet versus the current 500 mg racemate PZQ tablet (Cysticide) after single oral administration at a dose of 20 mg/kg of L-PZQ in healthy subjects under fed conditions.

Subjects were dosed in a 5-period crossover in different cohorts for logistical reasons with a 7 day washout between each administration of study drug. Treatments were:

(A) L-PZQ ODT formulation at 20 mg/kg dispersed in water, after a meal (Period 1 and 2) (n = 36).
(B) Current PZQ formulation (Cysticide) at 40 mg/kg given with water, after a meal uncrushed (Period 1 and 2) (n = 36).
(C) L-PZQ ODT formulation at 10 (C1) or 30 (C2) mg/kg (randomized 1–1) given dispersed in water, after a meal (Period 3, 4, and 5) (C1 n = 17, C2 n = 17).
(D) L-PZQ ODT formulation at 20 mg/kg given dispersed in water without a meal (Period 3, 4, and 5) (n = 35).
(E) L-PZQ ODT formulation at 20 mg/kg directly disintegrated in the mouth without water after a meal (Period 3, 4, and 5) (n = 36).

Doses in both studies were rounded to the nearest integer tablet size. So, for example, a 70 kg adult scheduled to receive 40 mg/kg of Treatment B, 2800 mg, would be rounded to 3000 mg (6 tablets).

The population to be included in each study consisted of male subjects age 18–55 years inclusive, and a body weight (BW) of 55–95 kg, who were certified as healthy by a comprehensive clinical assessment and fulfilled the inclusion and exclusion criteria. When a meal was to be administered with the dose in both studies, a standard high-carbohydrate meal was given consisting of a 100 g Breakfast cereal (All-Bran Flakes); 40 g Bread (Health Loaf/Granary); 250 g Milk (Low Fat/2% Fat, Fresh); 5 g Marmite, Yeast Extract; 10 g Sugar, White, Granulated. The meal contained ~ 75% carbohydrates of ~ 650 Calories. Serial plasma samples for pharmacokinetic analysis were collected from each subject in each period in each study. The primary endpoint for each study was the pharmacokinetic parameter AUC0-∞ of L-PZQ assessed in plasma.

All studies were conducted in accordance with the Declaration of Helsinki. Approval of the studies was done by the Medicine Control Council of South Africa and the Ethics Committee of the Faculty of Health Sciences, the University of the Free State, Bloemfontein.

### Population pharmacokinetic analysis using NONMEM

**Fig. 1:**
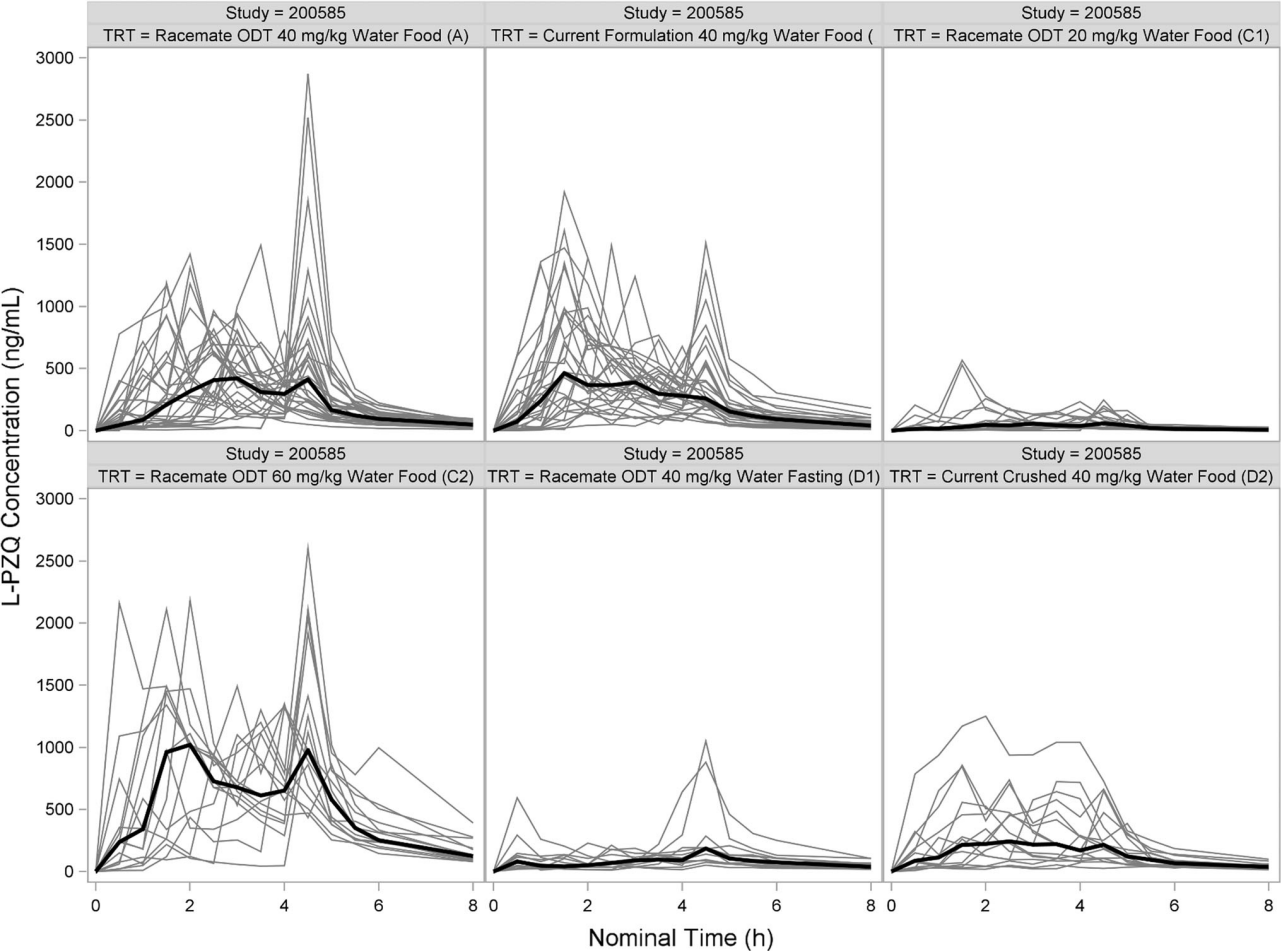
Spaghetti plot of L-PZQ concentration–time profiles after oral administration in Study 200585-001 stratified by treatment. Black line is the median concentration. Gray lines are individual subjects

**Fig. 2:**
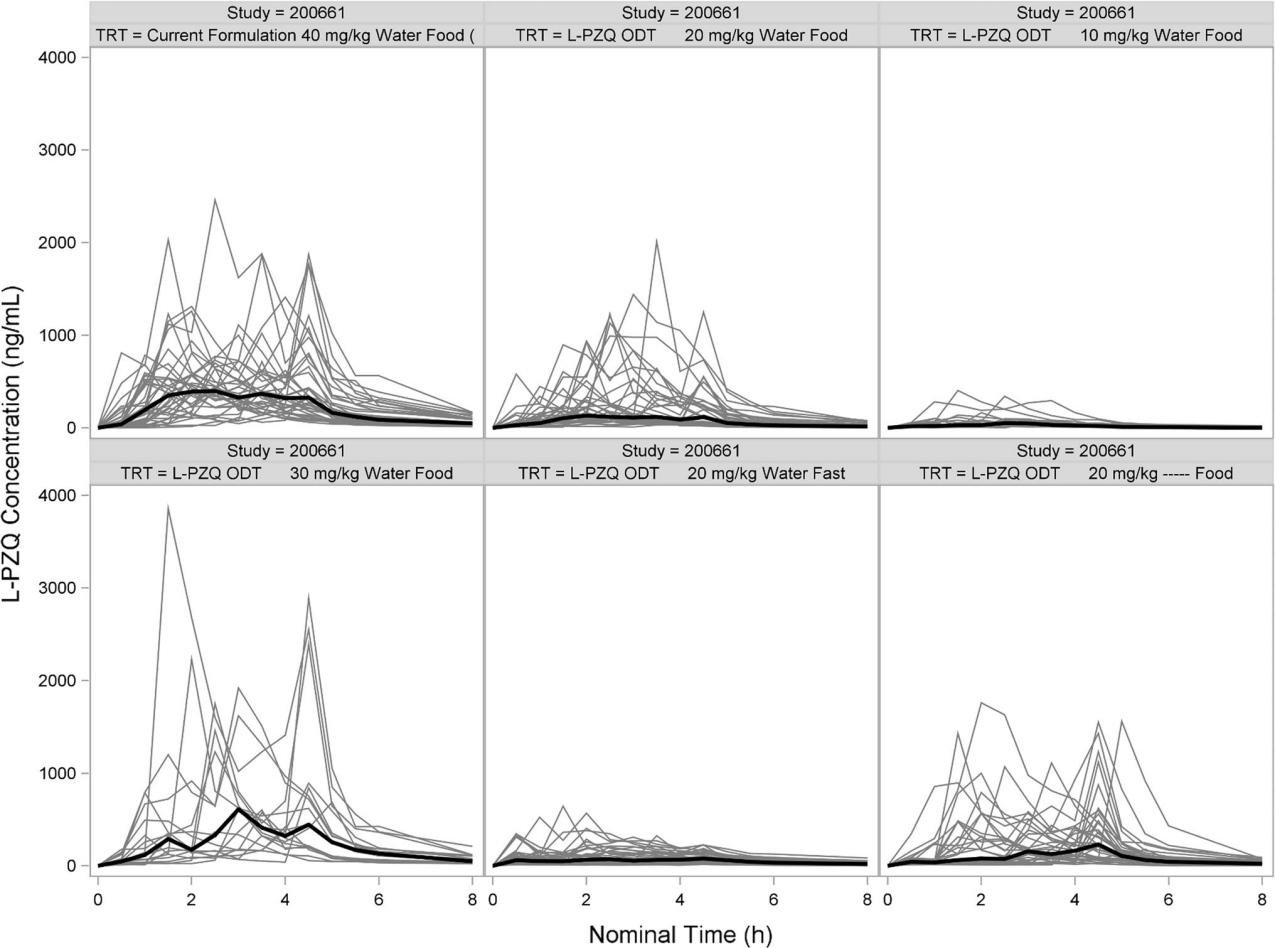
Spaghetti plot of L-PZQ concentration–time profiles after oral administration in Study 200661-001 stratified by treatment. Black line is the median concentration. Gray lines are individual subjects

Standard population pharmacokinetic (PopPK) methods and models using NONMEM (version 7.3, ICON Development Solutions, Ellicott City, MD) were used to analyze data from Study 200661-001 [7]. However, it became apparent during the analysis that the concentration–time profiles were not well-behaved and had multiple, irregular peaks (Figs. 1, 2). Dozens of models were examined with different absorption functions (split inputs, recycling, mixed first- and zero-order, etc.). Different estimation methods were tried (FOCEI, SAEM, etc.). These peaks caused severe problems and after weeks of intense effort, a suitable model could not be found. Hence, further model development using population methods was terminated.

### Population pharmacokinetics using linear mixed effects modeling

Since PopPK analysis using NONMEM failed, an alternative plan was devised. The concentration–time data comprised a single dose and the proposed Phase 2 pediatric study also was a single dose study. Therefore, it was concluded that a linear mixed effect model approach using the noncompartmental pharmacokinetic estimates would be a viable alternative to nonlinear mixed effects modeling of the concentration–time profiles and would lead to the same conclusions regarding proposed AUC in the pediatric population.

Noncompartmental analysis was done using Phoenix version 6.3 (Certara, St. Louis, MO). AUC(0–∞) was estimated using the linear up-log down trapezoidal method extrapolated to infinity. Cmax were estimated by direct examination of the data. Log-transformed noncompartmental estimates of AUC(0–∞), denoted AUC hereafter, were the dependent variables in the analysis. Linear mixed effects models were used to analyze the dependent variable as a function of the covariates [8]. This approach can be considered to be an extension of the power model for dose proportionality with the addition of covariates to the model

1 $$Ln\left( {AUC} \right) = Ln\left( {Dose} \right) + {\text{covariates}} .$$

All models were developed using the Mixed procedure in SAS for Windows (Version 9.3, SAS Institute, Cary NC). All models were fit using restricted maximum likelihood (REML). Fisher scoring was done if the initial model could not estimate the parameters. Each study was analyzed separately.

First, a full model with all covariates was fit to the data. For Study 200585-0001, the covariate list included: log-transformed L-PZQ dose administered (DOSE), period (PERIOD), log-transformed weight (WEIGHT), formulation (ODT, 0 = current, 1 = ODT), whether the tablet was crushed (CRUSH, 0 = no, 1 = yes), whether drug was administered with food (FOOD, 0 = no, 1 = yes), age of subject (AGE), and number of tablets administered (TABLETS). For Study 200661-0001 the covariate list included log-transformed DOSE, PERIOD, WEIGHT, ODT, drug taken with water (WATER, 0 = no, 1 = yes), FOOD, AGE, and TABLETS. Both intercept and log-transformed DOSE were treated as uncorrelated random effects. A simple residual covariance was assumed. Nonsignificant terms (p > 0.05 based on the Kenward-Rogers *T* test of the parameter estimate) were removed from the model until a parsimonious model with only statistically significant terms (p < 0.01) remaining in the model.

### Simulations and extrapolation to a pediatric population

The strategy for simulating exposures in African children (2 years to 18 years old) was as follows:

1. Model L-PZQ AUC data from Studies 200661-001 and 200585-001 separately using a linear mixed effects model based on noncompartmental estimates (Eq. (1)).
2. Establish Target AUC in Adults: Simulate L-PZQ exposure from 40, 50, and 60 mg/kg of current racemic PZQ formulation using model from Study 200661-001 using 1000 random resamples from 18 to 55 year olds weighing between 55 and 95 kg in the National Health and Nutritional Examination Status (NHANES) database [9]. These exposures are the reference exposures for comparison to children. Study 200585-001 would then be used as an external validation dataset in adults.
3. External Validation to Swiss TPH Study in *S. mansoni* infected Children (study details are given later in the section): Using the model developed for Study 200661-001, simulate L-PZQ AUC exposures following doses of 20, 40, and 60 mg/kg of the current formulation with food in an African pediatric population (2–5 years old) and compare the results to the Swiss TPH study [10].
4. Predict Doses in African Children to Match Adult Exposures: If Step 3 is successful then simulate and optimize the pediatric equivalent dose in African children for racemic ODT equivalent to current racemic formulation at 60 mg/kg in adults.
5. Predict Doses in African Children to Match Adult Exposures: If Step 3 is successful then simulate and optimize the pediatric equivalent dose for African children L-PZQ ODT equivalent to current racemic formulation at 40, 50, and 60 mg/kg in adults.

In Step 3, clearance in children (CL_children_) is expressed as

2 $$CL_{children} = CL_{adults} \left( {\frac{Weight}{{70{\text{ kg}}}}} \right)^{0.75} \times MFA .$$

where MFA is the degree of enzyme maturation. Hence, using the relationship AUC = F × Dose/CL and substituting Eq. (2) for CL, AUC in children can be modeled as:

3 $$AUC_{children} = \frac{{\left[ {{\text{Predicted AUC from Adult Model in Eq}} . { 1}} \right]}}{{MFA \times \left( {\frac{Weight}{{70{\text{ kg}}}}} \right)^{0.75} }} .$$

Modeling Cmax is problematic because no such equation exists. Hence, simulation of PZQ exposures in children was limited to AUC.

In order to simulate the AUC in African children, the weight of the children and the degree of enzyme maturation (MFA) relative to adults must be accounted for [11]. To account for weight, age was varied in the simulations and the weight of the African child was imputed using growth charts reported by the Liverpool School of Tropical Medicine [12], hereafter called the Liverpool dataset. MFA, which was provided by Simcyp within the Pediatric Praziquantel Consortium, was used to correct for enzyme maturation in very young children and was calculated on the basis of the ontogenies of each CYP isoform involved in the metabolism of L-PZQ and the fraction of L-PZQ clearance mediated by each CYP isoform determined from scaling in vitro metabolism data provided by Merck KGaA (data on file). Equation (4)–(8) describe the maturation of CYP isoforms involved in the metabolism of L-PZQ as a fraction of the adult abundance based on AGE:

4 $$CYP1A2 = \left\{ {\begin{array}{*{20}c} {0.24 + \frac{{1.47 \times AGE^{1.73} }}{{0.36^{1.73} + AGE^{1.73} }}} & {\text{if age < 2}} \\ {0.83 + 0.79\exp \left( { - 0.06\left( {AGE - 1.8} \right)} \right)} & {{\text{if age }} \ge 2} \\ \end{array} } \right.$$

5 $$CYP2C9 = 0.17 + \frac{{0.81 \times AGE^{0.53} }}{{0.0157^{0.53} + AGE^{0.573} }}$$

6 $$CYP2C19 = 0.3 + \frac{{0.68 \times AGE^{2.44} }}{{0.29^{2.44} + AGE^{2.44} }}$$

7 $$CYP3A4 = \left\{ {\begin{array}{*{20}c} {0.11 + \frac{{0.95 \times AGE^{1.91} }}{{0.64^{1.91} + AGE^{1.91} }}} & {{\text{if AGE < 2}} . 3} \\ {1.1 - 0.123\exp \left( { - 0.05\left( {AGE - 2.2} \right)} \right)} & {{\text{if AGE}} \ge 2. 3} \\ \end{array} } \right.$$

8 $$CYP3A5 = \left\{ {\begin{array}{*{20}c} {0.11 + \frac{{0.95 \times AGE^{1.91} }}{{0.64^{1.91} + AGE^{1.91} }}} & {{\text{if AGE < 2}} . 3} \\ {1.1 - 0.123\exp \left( { - 0.05\left( {AGE - 2.2} \right)} \right)} & {{\text{if AGE}} \ge 2. 3} \\ \end{array} } \right.$$

9 $$MFA = \left\{ {\begin{array}{*{20}c} \begin{aligned} 0.21 \times CYP1A2 + 0.23 \times CYP2C9 + 0.29 \times CYP2C19 + \hfill \\ 0.19 \times CYP3A4 + 0.08 \times CYP3A5 \hfill \\ \end{aligned} & {\text{if AGE < 25}} \\ 1 & {{\text{if AGE}} \ge 2 5} \\ \end{array} } \right. .$$

### External comparison to swiss TPH study

After completion of the analysis and all simulations of Steps 1 and 2, the results were compared to the observations from a randomized, single blind, parallel group Phase 2 study in African preschool (2–5 years old) and school-age (6–11 years old) *S. mansoni* patients [10]. In this study, children were randomized to receive a single-dose of placebo, 20, 40, or 60 mg/kg racemic praziquantel (Cesol 600 mg tablets, Merck KgA). Praziquantel was administered based on weight (to the nearest half tablet, respectively), which was measured during the physical examination before treatment. For the preschool children the tablets were crushed and mixed with 20-50 mL syrup-flavored water to mask the taste. A standardized food item (sandwich with butter or fishpaste) was provided before treatment.

Dried blood spots were collected at 0, 0.5, 1, 1.5, 2, 2.5, 3, 6, 8, 12, and 24 h after dosing and analyzed for L- and D-praziquantel concentrations (linear calibration range of 0.01–2.5 µg/mL), as well as its active metabolite trans-4-hydroxypraziquantel, using a validated, enantioselective LC–MS/MS method [13]. L-PZQ AUC results are presented here. These were compared to the predictions based on the linear mixed effect models developed in Steps 1 and 2. To make the comparisons valid, the raw noncompartmental results from that study were corrected for crushing of tablets (+20% multiplier, value taken from Treatment Arm D2 in Study EMR200585-001) and for use of dried blood spots as the bioanalytical matrix (+10% multiplier, value from [14]).

## Results

### Description of observed data

#### Study 200585-001

A total of 119 noncompartmental estimates of AUC from 32 subjects were available for analysis. Subjects ranged in age from 21 to 44 years with a mean of 29.0 years and from 53.5 to 91.6 kg in weight with a mean of 71.7 kg. The number of tablets administered (for both the current formulation and ODT formulation) ranged from 4 to 25 with a mean of 12.6. The number of subjects per treatment is shown in Table 1. Of all 32 (100%) healthy male subjects included in the trial 19 subjects (59.4%) were Black or African American, 11 subjects (34.4%) were White and 2 subjects (6.3%) were of other races.

**Table 1:** Number of Subjects for each treatment group by study

Study	Treatment	Formulation	Dose (mg/kg)	With water	Fast or fed	Crush tablets	Number of subjects
200585-001	A	Racemic ODT	40	Water	Food	No	31
	B	Current formulation	40	Water	Food	No	31
	C1	Racemic ODT	20	Water	Food	No	14
	C2	Racemic ODT	60	Water	Food	No	15
	D1	Racemic ODT	40	Water	Fasting	No	14
	D2	Current ODT	40	Water	Food	Yes	14
200661-001	A	L-PZQ ODT	20	Water	Food	No	36
	B	Current formulation	40	Water	Food	No	36
	C1	L-PZQ ODT	10	Water	Food	No	17
	C2	L-PZQ ODT	30	Water	Food	No	17
	D	L-PZQ ODT	20	Water	Fast	No	35
	E	L-PZQ ODT	20	–	Food	No	36

#### Study 200661-001

A total of 177 noncompartmental exposure parameters of AUC from 36 subjects were available for analysis. Subjects ranged in age from 19 to 47 years with a mean of 26.3 years and ranged from 55.2 to 87.8 kg in weight with a mean of 69.4 kg. The number of tablets administered ranged from 8 to 12 with a mean of 9.5. The number of subjects per treatment is shown in Table 1. Of all 36 healthy male subjects included in the trial, 27 subjects (75.0%) were Black or African American, 6 subjects (16.7%) were White and 3 subjects (8.3%) were of other races.

#### Swiss Tropical and Public Health Institute Study

A total of 28–33 AUC measurements per treatment group (95 children in total) ranging in age from 2 to 5 years old, treated with 20, 40 and 60 mg/kg, and ranging in weight from 8.0 to 22.3 kg were used for our analyses.

### Model development using nonlinear mixed effect modeling with NONMEM in study 200661-001

A total of 977 observations were available from 36 individuals from Study 200661-001. The concentration–time profiles for L-PZQ were erratic within and across subjects. Dozens of models using population pharmacokinetic methods were tested with a variety of absorption models. No acceptable base model could be found. The reasons for failure were myriad, but were predominantly due to unacceptable goodness of fit or convergence failure. As such, no model development was attempted for Study 200585-001.

### Modeling of AUC estimates using linear mixed effect models in SAS

#### Study 200585-001

**Fig. 3:**
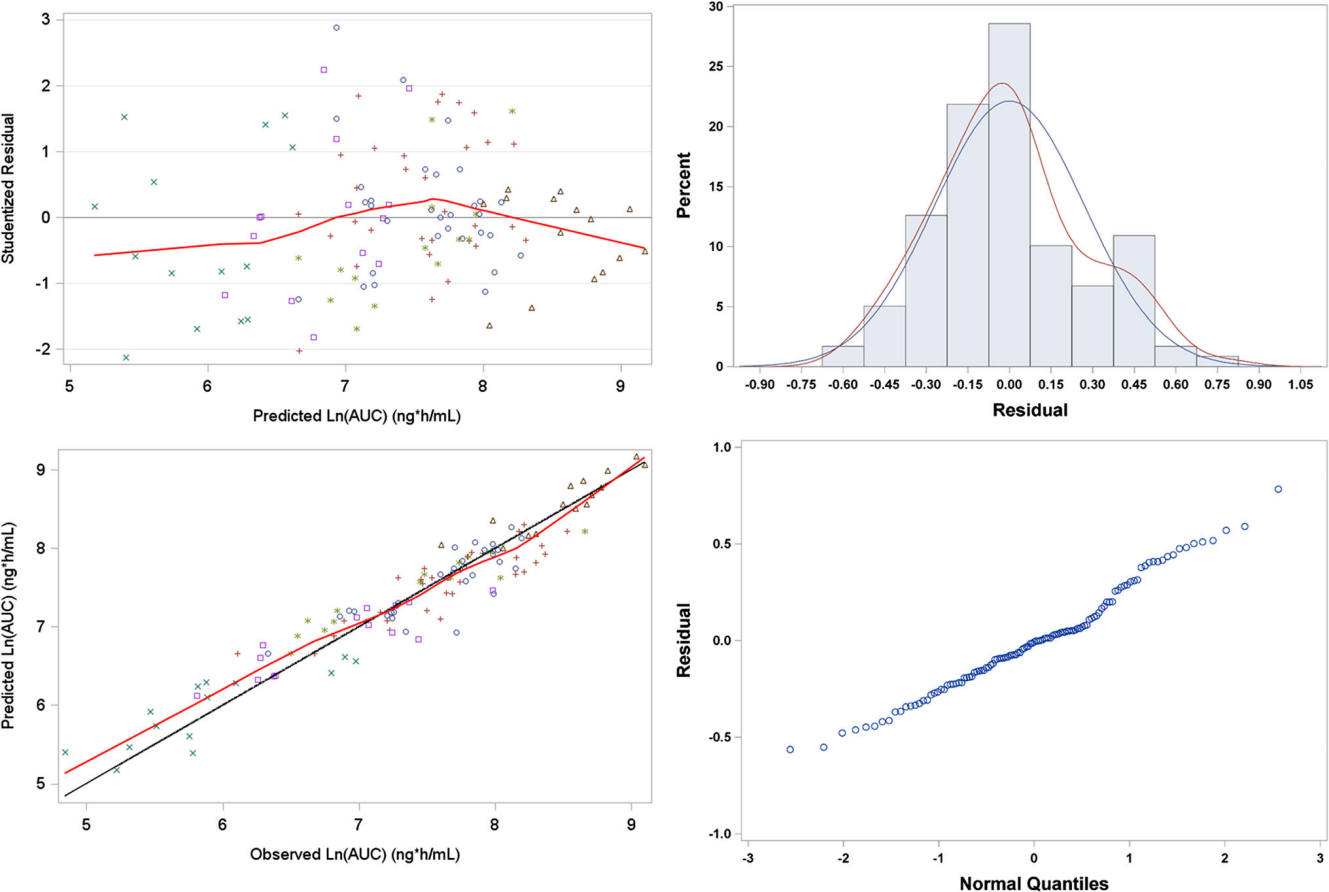
Goodness of fit plot for L-PZQ AUC model for Study 200585-001. Upper left: red line is the LOESS smooth to the data. Symbols are by treatment. Upper right: blue line is standard normal distribution, red line is kernel smooth to the empirical data. Lower left: black line is line of unity, red line is LOESS smooth. Symbols are by treatment. Lower right: QQ plot of residuals; blue line is theoretical normal distribution line

The Akaike Information Criterion (AIC) for the full model with all covariates was 154.5. The best model, which had an AIC of 139.2, was one where only log-transformed DOSE and FOOD were included in the model. All other terms were not statistically significant (p > 0.01). The parameter estimates for the AUC model are shown in Table 2. Goodness of fit plots for the model are shown in Fig. 3. The best fit AUC model was:

**Table 2:** Parameter estimates from best linear mixed effect models For L-PZQ AUC in Healthy volunteers enrolled in studies 200585-001 and 200661-001

Study	Parameter	Estimate	Standard error	T test	p value
200585-001	Intercept	− 9.99	0.850	− 11.76	< 0.0001
	Ln-DOSE	2.30	0.116	19.9	< 0.0001
	Food	0.811	0.0956	8.49	< 0.0001
	Var(Intercept)	0.209			
	Residual Variance	0.0972			
200661-001	Intercept	− 7.52	0.930	− 8.09	< 0.0001
	Ln-DOSE	2.01	0.126	15.98	< 0.0001
	ODT formulation	− 0.81	0.0746	− 10.85	< 0.0001
	Food	0.62	0.0754	8.17	< 0.0001
	Var(Intercept)	0.372			
	Residual Variance	0.148

10 $$Model \, Ln\left( {AUC} \right) = - 9.99 + 2.3 \times Ln\left( {\text{L - PZQ Dose in mg}} \right) + 0.81 \times FOOD$$

which in the original domain can be written as:

11 $$\begin{aligned} Ln\left( {AUC{\text{ in ng*h/mL}}} \right) = & - 9.99 + 2.3 \times Ln\left( {\text{L - PZQ Dose in mg}} \right) + 0.81 \times FOOD \\ AUC = & \frac{{DOSE^{2.3} \exp \left( {0.81} \right)^{FOOD} }}{{\exp \left( {9.99} \right)}} \\ AUC = & \frac{{\left( {DOSE} \right)^{2.3} \times 2.25^{FOOD} }}{22026} \\ \end{aligned} .$$

Equation (11) can also be expressed in terms of apparent oral clearance. It was assumed that the effect of Food would be manifest through its effect on F. Equation (11) was then rewritten as

12 $$AUC = \frac{{\left( {DOSE} \right)^{2.3} }}{{\frac{CL}{F}}} = \frac{{\left( {DOSE} \right)^{2.3} }}{{\frac{22026}{{2.25^{FOOD} }}}} .$$

These results showed that the presence of food increased AUC by 125% and that the dose-AUC relationship was supraproportional. They further showed that none of the other available covariates (age, weight, number of tablets, etc.) significantly influenced AUC.

#### Study 200661-001

The AIC for the full model with all covariates was 288.2. The best model, which had an AIC of 269.0, was one where only log-transformed DOSE, ODT formulation, and FOOD were included in the model. All other terms were not statistically significant (p > 0.01). The parameter estimates in the AUC model are shown in Table 2. Goodness of fit plots for the model are shown in Supplemental Fig. 1. The best fit AUC model was:

13 $$Model \, Ln\left( {AUC} \right) \, = - 7.52 + 2.0 \times Ln\left( {\text{L - PZQ Dose}} \right) - 0.81 \times ODT + \, 0.62 \times FOOD$$

Which in the original domain can be written as:

14 $$\begin{aligned} Ln\left( {AUC} \right) & = - 7.52 + 2.0 \times Ln\left( {{\text{L - PZQ Dose in mg}}} \right) - 0.81 \times ODT + 0.62 \times FOOD \\ AUC & = \frac{{DOSE^{{2.0}} \exp \left( {0.62} \right)^{{FOOD}} }}{{\exp \left( {7.52} \right)\exp \left( {0.81} \right)^{{ODT}} }} \\ AUC & = \frac{{\left( {DOSE} \right)^{2} \times 1.86^{{FOOD}} }}{{1845 \times 2.25^{{ODT}} }} \\ \end{aligned}$$

These results showed that the presence of food increased AUC by 86%, that there was a 55% decrease in AUC with the L-PZQ ODT formulation, and that the dose-AUC relationship was supraproportional. They further showed that none of the other available covariates (age, weight, number of tablets, etc.) influenced AUC.

### External comparison to swiss TPH study

Aggregate noncompartmental pharmacokinetic data from the Swiss TPH study were used from 95 patients. The results for total AUC stratified by total dose are shown in Table 3.

**Table 3:** Comparison of observed and simulated L-PZQ AUC estimates

Dose (mg/kg)	Observed study 200661-001	Simulated using model 200661-001	STPHI study
20			2730 (489–17871)
40	2066 (660–6746)	2164 (599–7893)	3256 (726–7987)
50		3474 (1011–11666)	
60		5101 (1427–17235)	5567 (855–22822)

Data are reported as mean (range)

### Extrapolation and Simulation of pediatric exposures

L-PZQ exposures in adults were simulated at doses of 40, 50, and 60 mg/kg with the current racemic formulation when administered with food and water using the NHANES adult database and the model from Study 200661-001 (Step 2 of the Simulation). Because of high variability of the tails of the distribution, the upper and lower tails were trimmed by 10% prior to data summarization. The observed and trimmed simulated results are presented in Table 3. Note that the observed target AUC in adults from 200661-001 study was 2066 ng*h/mL. The model did a reasonable job of predicting total AUC and was within 20% relative error for the median prediction.

The next step (Step 4) was to simulate doses of 20, 40, and 60 mg/kg of the current formulation with food using an African pediatric population and compare the results to the Swiss TPH study. Supplemental Fig. 2 presents a band plot of weight as a function of age in Africans and its comparison to Western subjects in the US NHANES database. Although the growth trajectories are similar, the weight of Africans is smaller than Western population. This importance of this difference will manifest itself later in the dose requirements for Africans requiring smaller doses than their Western counterparts. Supplemental Fig. 3 presents a scatter plot of the observed doses used in the clinical studies compared to the simulated doses based on the Liverpool weight data. The simulated subject weights using the Liverpool dataset were similar to the observed subject weights in the Swiss TPH study confirming the validity of the Liverpool dataset to estimate age-specific weights in African children.

**Fig. 4:**
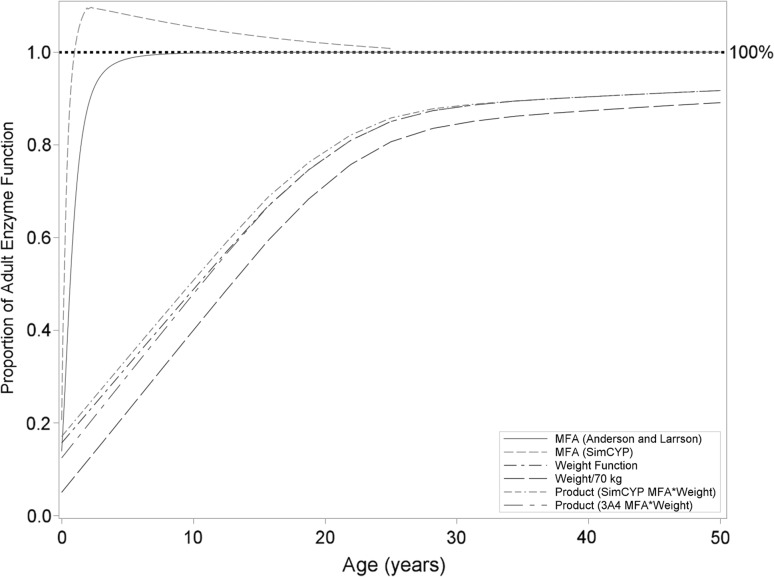
Effect of allometric weight and MFA, as calculated by two different methods (SimCYP method used in this analysis and CYP3A4 function reported by Anderson and Larrson [15], on clearance in Africans

Figure 4 presents a series plot of the effect of MFA on clearance in children (using the MFA equation used in this analysis compared to a published CYP3A4 MFA function published by Anderson and Larsson [15] as a function of age. Although the effect of MFA tends to plateau around 2 years of age, the overall effect on clearance, as reflected by the product of MFA and weight, doesn’t asymptote until well into adulthood and is predominantly linear in nature until then. Based on the results of the modeling of noncompartmental parameters from Study 200661-001, Eq. (14) was modified to incorporate the allometric effect of weight and effect of MFA in young children:

15 $$AUC_{children} = \frac{{\left( {\text{L - PZQ DOSE}} \right)^{2} \times 1.86^{FOOD} }}{{1845 \times 2.25^{ODT} \left( {\frac{\text{Weight}}{{70{\text{ kg}}}}} \right)^{0.75} \times MFA}} .$$

**Fig. 5:**
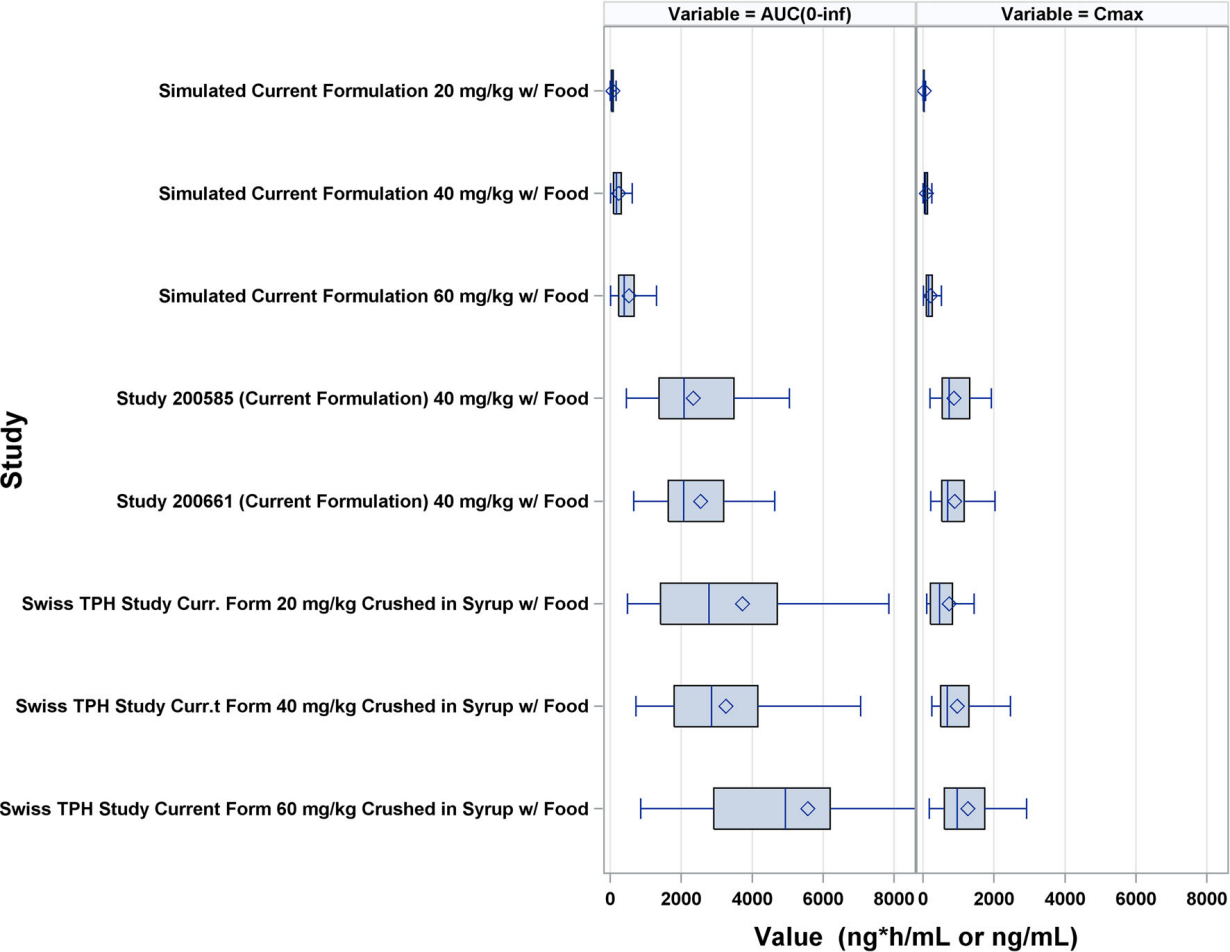
Forest plot comparing the observed L-PZQ AUC in Study 200585-001, Study 200661-001, and the Swiss Tropical and Public Health Institute Study to simulated AUCs based on the model developed using Study 200661-00. Each box is the 1st and 3rd quartile. The middle line in the box is the median (2nd quartile). The diamonds are the mean. The whiskers are 1.5 times the inter-quartile range

Figure 5 presents the observed AUC in Study 200585-001, 200661-001, and the Swiss TPH study compared to simulated values under the model developed using Study 200661-001. The model predicted values were significantly less than observed values from the Swiss TPH study. Simulated median AUC was underpredicted by ~ tenfold compared to observed values from the Swiss TPH study. At this point further modeling and simulation efforts ceased because, despite our best efforts, a suitable model that could reliably extrapolate to a pediatric population could not be developed.

## Discussion

### Failure analysis—post-mortem

It was clear from the results of the Swiss TPH study that the predicted exposures in African children extrapolated from the adult model were significantly lower than the observed results of the Swiss TPH study and that a dose of 40 mg/kg of the current formulation in African children would provide equivalent exposure as 40 mg/kg of the same formulation in adult Western healthy volunteers. The question is why? Either the model was “wrong” and the predictions from the model were too low or the model was “right” and the observed data were higher than expected. Were there clues that might have told us to be wary of the simulation results? Maybe. In hindsight.

After it was realized that the modeling results were not successful, a root cause failure analysis (RCFA) was undertaken to look for possible reasons for the failure. RCFA has its origin in the NASA program to understand why rockets failed in their launches [16]. One definition of root cause analysis is [16]:

The primary aim of root cause analysis is: to identify the factors that resulted in the nature, the magnitude, the location, and the timing of the harmful outcomes (consequences) of one or more past events; to determine what behaviors, actions, inactions, or conditions need to be changed; to prevent recurrence of similar harmful outcomes; and to identify lessons that may promote the achievement of better consequences. (“Success” is defined as the near-certain prevention of recurrence).

A brainstorming session was held and a number of factors were identified as possible causes:

1. The model was “wrong” and predicted exposures were lower than expected, or
2. The model was “right” but the observed Swiss TPH exposure data were higher than expected because of:
  - Differences in the study population (healthy volunteers vs patients);
  - Differences between crushed tablets and intact tablets, number of tablets administered, or solubility saturation;
  - Differences in the meal composition;
  - Differences in bioavailability between adults and children; and/or
  - Differences in biological matrix (plasma vs dried blood spot).

**Fig. 6:**
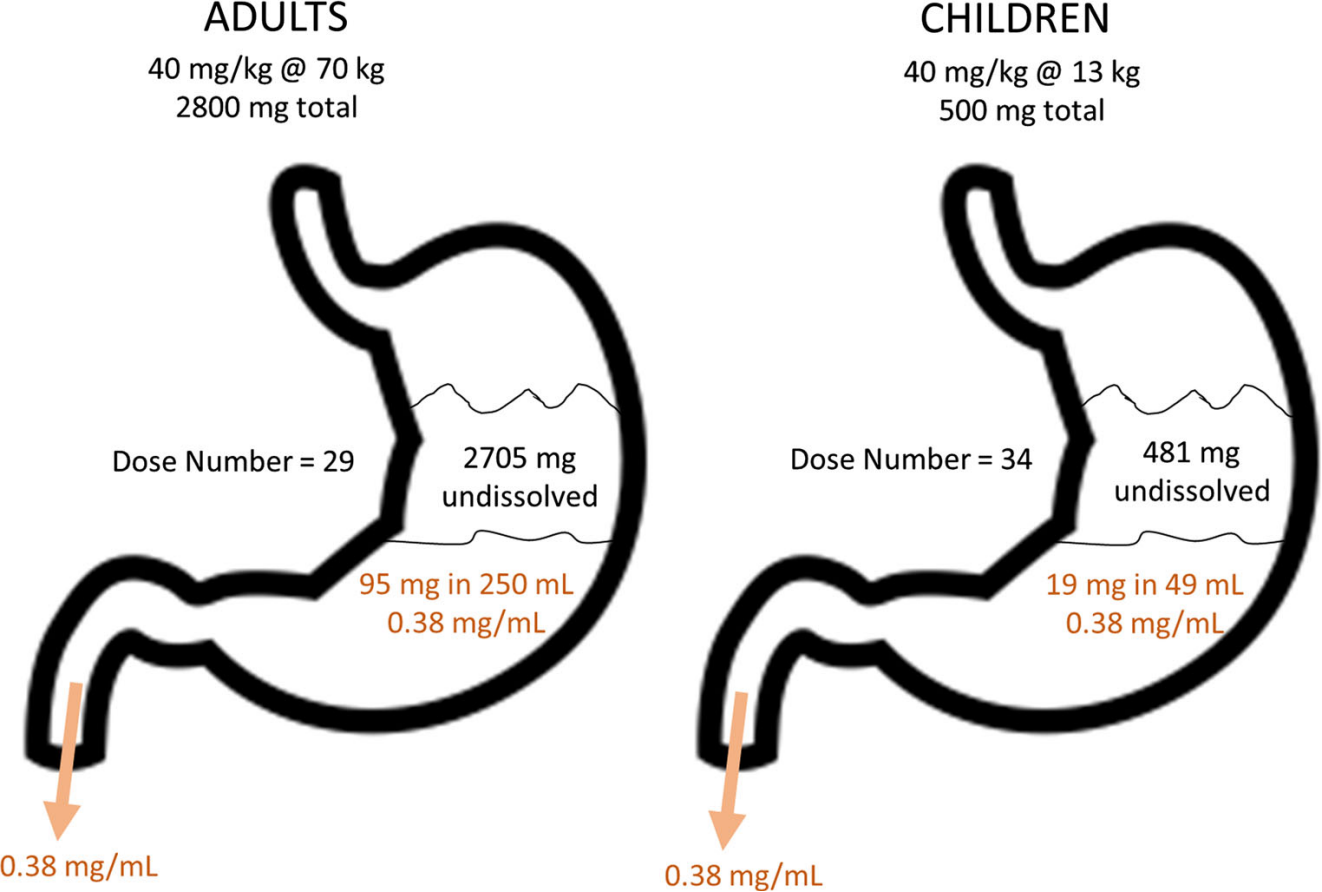
Cartoon of drug dissolution in the stomach of adults and children. It should be noted that dissolution can also take place in the intestine
- There were also differences in the meal composition between the Western adult studies and Swiss TPH study that may have led to a difference in predicted exposures. The Western adult studies used a standard high-carbohydrate meal, while the Swiss TPH study used a sandwich with butter or fishpaste, which is more towards a high fat meal. Castro et al. [26] showed that PZQ pharmacokinetics were dependent on the composition of the meal. A standard high fat meal increased AUC by 172% and Cmax by 212% compared to the fasting state, but a high carbohydrate meal increased AUC and Cmax even more, 298 and 484%, respectively. It is possible that differences in meal type between studies could account for some of the differences between the observed Swiss TPH results and predicted exposures.
- Another reason for the discrepancy could be related to differences between adults and children in oral absorption and first pass metabolism. It is assumed in any pediatric extrapolation that oral bioavailability (F) is the same in adults and children such that apparent oral clearance (CL/F) scales with weight. This works only if F is a constant between adults and children. Often this assumption is left unsaid in manuscripts and in presentations but it is a critical assumption. The correction using weight and maturation factors during the extrapolation process are for changes in total systemic clearance (CL) with age. There are no corrections for F with age, despite there being known differences in young children, particularly very young children less than a year of age [27]. Is F a constant in adults and children for PZQ? Patzschke et al. [28] showed that following a standard breakfast (200 mL water, 1 roll with margarine, boiled ham, and a cup of coffee), renal excretion after a radioactive dose of 46 mg/kg PZQ was 80 ± 6%. Therefore, the fraction of dose absorbed, either as parent drug or metabolites, must be at least 80%. Hence, while there might be some role for differences in F, it seems unlikely to explain the discrepancy. Related to this explanation is another possibility that adults may have a clearance pathway that school-age children do not. Although CYP pathways mature by 2 years of age, it’s possible there is an extra-hepatic pathway that hasn’t fully matured yet. There is no experimental evidence for this nor are there examples such pathways exist, so this possibility cannot be confirmed. In total, differences in first pass metabolism and oral absorption seem an unlikely cause to explain the simulation differences.
- Another explanation could have been that the Swiss study used dried blood spots as the sample matrix and the Western studies used plasma. This was quickly ruled out because the analytical methods were both validated and were largely interchangeable with only a 10% difference in measured concentrations (plasma was slightly higher than DBS) [14].

Each factor was examined and either accepted or rejected as a possible factor:

- Could the model have been “wrong”? Could the discrepancy between simulated and observed exposures be explained by an inadequate model? Without going into the semantics of all models being wrong, could the extrapolation of adults to children resulted in predicted exposures that were too low? It should be pointed out that this was the first and immediate reason team members used to explain the discrepancy because the modeling approach that was used was not a standard approach and because “it’s a model.” The typical pediatric extrapolation approach is to first model the concentration–time profiles using PopPK and then extrapolate to children using an allometric scaling factor on clearance to account for differences in weight and, in really young children, to use a maturation factor to account for differences in enzyme immaturity. The approach herein modeled the AUC directly, and by extension, clearance indirectly since an adequate PopPK model could not be developed due to the erratic nature of the concentration–time profiles in adults. Since only a single dose of PZQ is given therapeutically, these approaches should have produced equivalent results. Indeed, the LMEM was very good at predicting the observed data in adults across all treatment arms. It may be that the traditional allometric scaling correction and maturation function used in this analysis do not apply to African children. There is only 1 report in the literature that we could find related to pediatric extrapolation in an African population. Zvada SP et al. [17] reported on the successful extrapolation of rifampicin, pyrazinamide, and isoniazid exposures in African children with tuberculosis. For those 3 drugs the authors used the standard allometric equations to scale down clearance from adults to children and used a maturation function to account for age-related differences in children less than a year old. The maturation function was defined by a Hill model that starts from zero and asymptotes to an adult value of 1, and in which the values of TM_50_ (the age at which maturation is 50% of the adult value) and Hill (the slope coefficient) were fitted to the available data [18]. Our analysis used similar extrapolation methods as Zvada et al., although our maturation function was different than theirs, so the likelihood that extrapolation functional differences explain the discrepancy is low.
  We also questioned whether the population-extrapolation approach is universally successful. It could be that the PopPK approach, which works often in practice, has led to an unreasonable expectation that, due to publication bias, this approach will work uniformly for all drugs in every case. Certainly there is this impression in the literature as any example of extrapolation failure could not be found on PubMed and it does seem unlikely that any single method would work universally in every case for every drug. Bonate and Howard [19] stated that the predictability of human pharmacokinetic parameters from allometric scaling of animals to man was overly optimistic because of a positive publication bias in the literature and yet here we are again, almost 20 years later, suggesting that allometric scaling of adult data to pediatrics might have the same bias, but may still be correct.
  Another suggestion was that the simulations were based only on the model developed from the 200585-001 data and that perhaps a model using all the data from both studies would result in better predictions. A secondary analysis was completed after this suggestion was made and the results are shown in Supplemental Model 1. Using a combined model did not improve predictions—there was still a wide discrepancy between observed and predicted exposures. In the end, it’s impossible to say with certainty if the discrepancy was due to model failure without another external dataset to validate against.
- The other reason for the discrepancy could be broadly categorized as the model was “correct” but the observed data in African children was higher than expected. There were many differences in the study design and populations used to develop the model and the target population the model would be extrapolated to. The model was built using data from a well-controlled, Phase 1 adult, Western South African population and was being extrapolated to a pediatric infected African population. Could the differences be explained by race? It seems unlikely. In Study 200585-001, 59% of the subjects were Black, while in Study 200661-001 the number was 75% Black. While the Phase 1 results were largely based on a Black study population, it seems reasonable to generalize the results to all Black Africans on face-value. A second factor considered were differences in subject weight. The simulations used a dataset developed by the University of Liverpool to simulate a given weight based on a given age range. If the weight ranges in the Liverpool dataset were not representative of the weight ranges used in the Swiss TPH study then different exposures could have been obtained. But examination of the simulated weights and observed weights in the Swiss TPH study revealed them to be similar (Supplemental Fig. 3), ruling this out as a possibility.
  A third factor considered was that the Western adult studies were in healthy volunteers while the Swiss TPH study was in infected children. Mandour et al. [20] studied the differences in PZQ pharmacokinetics in healthy volunteers and Sudanese schistosomiasis patients with different grades of liver impairment using the Distocide^®^ and Biltricide^®^ formulations. Large differences in exposure were seen related to the degree of liver impairment. For example, AUC was 4- to 5-times higher in severely impaired patients (Child–Pugh C) than healthy volunteers after administration of 40 mg/kg Biltricide (15928 vs. 3823 ng*h/mL, respectively). Watt et al. [21] reported similar finding in Filipino patients with a disease-dependent increase in exposure with severe (AUC_24_ = 37.8 μg*h/mL) > moderate (AUC_24_ = 22.9 μg*h/mL) > unapparent (AUC_24_ = 8.9 μg*h/mL) hepatic disease. Since schistosomiasis causes periportal fibrosis and liver cirrhosis due to deposition of eggs in the small portal veins [22], an increase in AUC in the disease state seems entirely reasonable, although the disease in young children is generally not accompanied by liver abnormalities. In addition, 80% of preschool children analyzed in the Swiss TPH study harbored light infections [23], hence liver abnormalities are not expected.
  And lastly, it was considered was that PZQ absorption is sensitive to gastrointestinal (GI) pH. Sammon et al. [24] showed that in rural South African adults the mean 24 h stomach pH was 2.84 and at night was as high as 3.7, which was considerably higher than historical data in Western subjects. Reaching pH levels seen after cimetidine administration (pH 3.1–6, cimetidine package insert), higher basal stomach pH in African children may have contributed to higher than expected absorption and higher than expected exposure in the Swiss TPH study. Therefore, there were differences in the patient population that could explain part of the differences in the predicted exposures simulations.
- Another possible reason for the difference could have been differences in absorption and bioavailabilty due to crushing, the number of administered tablets, or solubility saturation. In the Swiss TPH study, the PZQ tablets were crushed prior to administration in preschool age children. In Study 200585-001, crushing decreased the L-PZQ AUC by 18% (90% CI: 31–2%) for the marketed formulation Cysticide. Hence, crushing decreased absorption, it did not increase it. Therefore, the effect of crushing could not account for the larger than expected exposures seen in the Swiss TPH study.
  Maybe the difference could have been due to the number of tablets administered? In Study 200585-001, a large number of tablets were administered, 7–35 for the ODT formulation and 4–8 with Cysticide. In Study 200662-001, 5–7 Cysticide tablets and 4–17 ODT tablets were administered. For the Swiss study, 1–3 tablets were administered. Administration of a large number of tablets could result in a different dissolution profile and change the oral absorption of the drug. This hypothesis was put forth early as a reasonable explanation. However, statistical analysis of the 200585-001 and 200662-001 data did not detect any effect of number of tablets administered, i.e., the number of administered tablets did not affect exposure. Therefore, this seems an unlikely reason for the difference in exposures.
  Related to this was the hypothesis that PZQ saturation in GI fluids and its effect on absorption was the reason for the difference. PZQ is a Biopharmaceutics Class System (BCS) II drug meaning it has high permeability, low solubility and dissolution is the rate-limiting step in the absorption of PZQ. The therapeutic dose of 40 mg/kg in a 70 kg adult is around 3000 mg, which is quite high. With a water solubility of 0.38 mg/mL (https://www.drugbank.ca/drugs/DB01058) and a fasted state simulated intestinal fluid (FaSSIF) solubility of 0.26 mg/mL (Fagerberg et al. 2015), this means that in 70 kg adults receiving a dose of 2800 mg, drinking a water volume of 250 mL, only 95 mg of drug is in solution at any given time with more than 97% of the dose initially not in solution (Fig. 6). In a 5 year old child weighing 13 kg who received a dose of 500 mg drinking a water volume of 50 mL (Shawahna R 2016), 19 mg PZQ is in solution (~ 3% of the dose). One hypothesis assumed that with the bulk of drug being undissolved in the stomach and since water in the GI tract is not uniform but found in pockets [25], the erratic concentration–time profiles with multiple peaks could be explained by drug solubilization in water pockets and subsequent absorption, or by non-documented additional water intake by the subjects. But again, since the 80% of the drug is absorbed, despite a large amount of initially undissolved drug, sink conditions on the basolateral side of the enterocytes during intestinal absorption would provide an explanation for the amount of drug-related material absorbed, and thus it seems unlikely that an increase in F in children would result in the kind of exposure discrepancy that was observed.

## Conclusions

Because of the erratic nature of the concentration—time profiles, a suitable population pharmacokinetic model could not be developed using standard nonlinear mixed effect models. Using linear mixed effect modeling of the noncompartmental estimates for AUC a suitable predictive model could be developed, which produced parameter estimates consistent with the statistical analysis of the noncompartmental estimates. However, when this model was used to extrapolate to a pediatric population, the simulated exposures were ~ tenfold lower compared to results obtained from a clinical study in the population of interest (the Swiss TPH study). A post-mortem afterwards suggested possible reasons for this difference, with differences in the meal composition and study populations being of sufficient magnitude to explain the discrepancy.

Root cause analysis highlighted a number of important considerations that are not often made or reported in the literature. First, pediatric extrapolation likely has a publication bias—negative studies where the extrapolation has failed are not reported. Journals need to encourage publication of failed pediatric extrapolations so that modelers can learn from them and not make the same mistakes next time. Second, a very important assumption made in pediatric extrapolation is constant oral bioavailability from adults to children. The allometric scaling equation and maturation function were designed to work for scaling total systemic clearance assuming absorption is a constant in adults and children. This may or may not be the case for every drug. Shawahna [29] showed that the Biopharmaceutics Classification System Class can change from adults to children due to differences in gastric volume and, if this is the case, it seems likely that bioavailability may change as well. This factor should be at least considered in any pediatric extrapolation.

Study- and population-related differences seem the most likely cause to explain the difference between the observed and simulated data (Table 4). Two of them, patients versus healthy volunteers and differences in meal composition, were of sufficient magnitude to suggest that these differences seen in published accounts could explain the discrepancy. Could all or some of these factors have played a role? Yes. But it is impossible to identify with any certainty the reasons for the discrepancy. The reasons do lead to possible future studies and hypotheses to be tested.

**Table 4:** Root Cause Analysis

Reason	Possible magnitude of effect	Likelihood to explain discrepancy
*Model was “wrong”*		
Use of LMEM was not appropriate	Small	Unlikely (but unknown for sure)
Allometric scaling was inappropriate	Unlikely	Unlikely
Wrong maturation factor used	Unlikely	Unlikely
Did not use all the data	Small	Unlikely
Differences in infected patients and healthy volunteers not accounted for in model	Small to Large	Possible to Likely
*Study results were higher than normal*		
Racial differences (Western vs. African, Caucasians vs Blacks)	Small	Unlikely
Weight differences	Small	Unlikely
Healthy volunteers vs infected patients	High	Likely
Differences in meal types	High	Likely
Differences in stomach pH between Western and African patients	Small	Possible
Extrahepatic metabolic pathway in adults not seen in children	Small to moderate	Unlikely
Crushing of tablets in Swiss TPH study	Small	Possible
Differences in oral bioavailability between adults and children	Small	Possible
Differences in analytical methods	Small	Unlikely
Number of tablets administered	Small	Unlikely
Differences in PZQ saturation in GI tract between adults and children	Small	Unlikely

While the idea of a root cause analysis to identify the reasons for model failure is useful, one must be careful not to put too much weight in their results because of confirmation bias. We know that the simulated and observed exposures were off by about tenfold. So going back and looking for factors that might lead to a tenfold increase in exposure and bring the exposure predictions in agreement with the observed Swiss TPH results is self-confirmatory. In other words, we look for reasons that are in agreement with our tenfold discrepancy and rule out those that don’t increase agreement between observations and predictions. Nevertheless, the root cause analysis did identify some factors and assumptions that might be useful to test and control in future pediatric extrapolations. These include a careful examination of differences in pharmacokinetics between healthy volunteer and patients, differences in drug administration and possible changes in BCS Class between adults and children, and an examination that oral bioavailability is a constant between adults and children.

## Electronic supplementary material

Below is the link to the electronic supplementary material.

Supplementary material 1 (DOCX 118 kb)

Supplementary material 2 (PNG 33 kb)

Supplementary material 3 (PNG 72 kb)

Supplementary material 4 (PNG 30 kb)
