# Supplementary material for: Extrapolation of praziquantel pharmacokinetics to a pediatric population: a cautionary tale
Source: J Pharmacokinet Pharmacodyn. 2018 Sep 14;45(5):747–62. doi: 10.1007/s10928-018-9601-1 (PMC6182730; doi:10.1007/s10928-018-9601-1)
Supplement: Supplementary file 1 — Supplementary material 1 (DOCX 118 kb) [file 10928_2018_9601_MOESM1_ESM.docx]

# Supplemental Model 1

A criticism of the analysis was the initial decision to model the 200585-001 and 200660-001 data separately and then base the pediatric extrapolation solely on Study 200585-001. A later analysis was conducted wherein a combined data model was developed using the same methods (with one exception) and procedures as before and then base the simulation on the joint model. In order to account for the differences in ODT formulations between studies, ODT was added as categorical factor in the linear mixed effects model (0=current formulation, 1=Racemic ODT, 2=L-PZQ ODT), as was STUDY (0=200585, 1=200660). Under the joint model, a total of 296 observations were available from 32 subjects in Study 200585-001 and 36 subjects from Study 200660-001.

***AUC:*** The AIC for the full model with all covariates was 416.4. The best model, which had an AIC of 409.2, was one where only log-transformed DOSE, ODT, and FOOD were included in the model. All other terms were not statistically significant (p > 0.01), including STUDY. The parameter estimates for the AUC model are shown in Supplemental Model Table 1. Goodness of fit plots for the model are shown in Supplemental Model Figure 1. The best fit AUC model was:

| $Model\text{ }Ln\left(AUC\right)\text{ }=-8.54+2.13\times Ln\left(\text{Dose}\right)+\text{ }0.667\times FOOD+\left\{\begin{matrix} 0 & \text{Current formulation} \\ -0.0794 & \text{L-PZQ ODT} \\ -0.776 & \text{Racemic ODT} \end{matrix}\right.$ |  |
| --- | --- |

In a comparison to the fixed effects of the two individual study-specific models, the fixed effect estimates were quite comparable. What wasn’t comparable was the intercept, which in the combined data model was a weighted mean of the intercepts from the study-specific values.

***Extrapolation and Simulation of Pediatric Exposures:*** A repeat of the simulations using the combined models showed similar results to results obtained using the study-specific model (Supplemental Model Figure 2). These results showed that a combined model did not improve the accuracy of the predictions and that there was still a discrepancy between the observed Swiss TPH data and the predicted data.

| **Supplemental Model Table 1: Parameter Estimates from Best Linear Mixed Effect Models Under the Combined Data Model for L-PZQ AUC** | | | | | |
| --- | --- | --- | --- | --- | --- |
| **Parameter** | **ODT** | **Estimate** | **Standard Error** | **T-test** | **p-value** |
| Intercept |  | -8.54 | 0.653 | -13.08 | < 0.0001 |
| Ln-DOSE |  | 2.13 | 0.0884 | 24.14 | < 0.0001 |
| ODT | Current | 0 | --- |  |  |
|  | Racemic | -0.079 | 0.0690 | -1.15 | 0.2504 |
|  | L-PZQ | -0.776 | 0.0657 | 11.87 | < 0.0001 |
| Food |  | 0.668 | 0.0601 | 11.13 | < 0.0001 |
| Var(Intercept) |  | 0.290 |  |  |  |
| Residual Variance |  | 0.130 |  |  |  |

**Figure 1 For Supplemental Model**

| 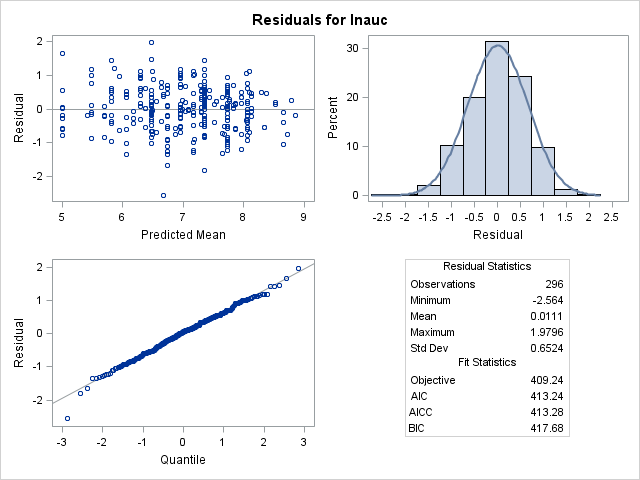 |
| --- |
| Goodness of fit plots for L-PZQ AUC under the combined data model. |

**Figure 2 For Supplemental Model**

| 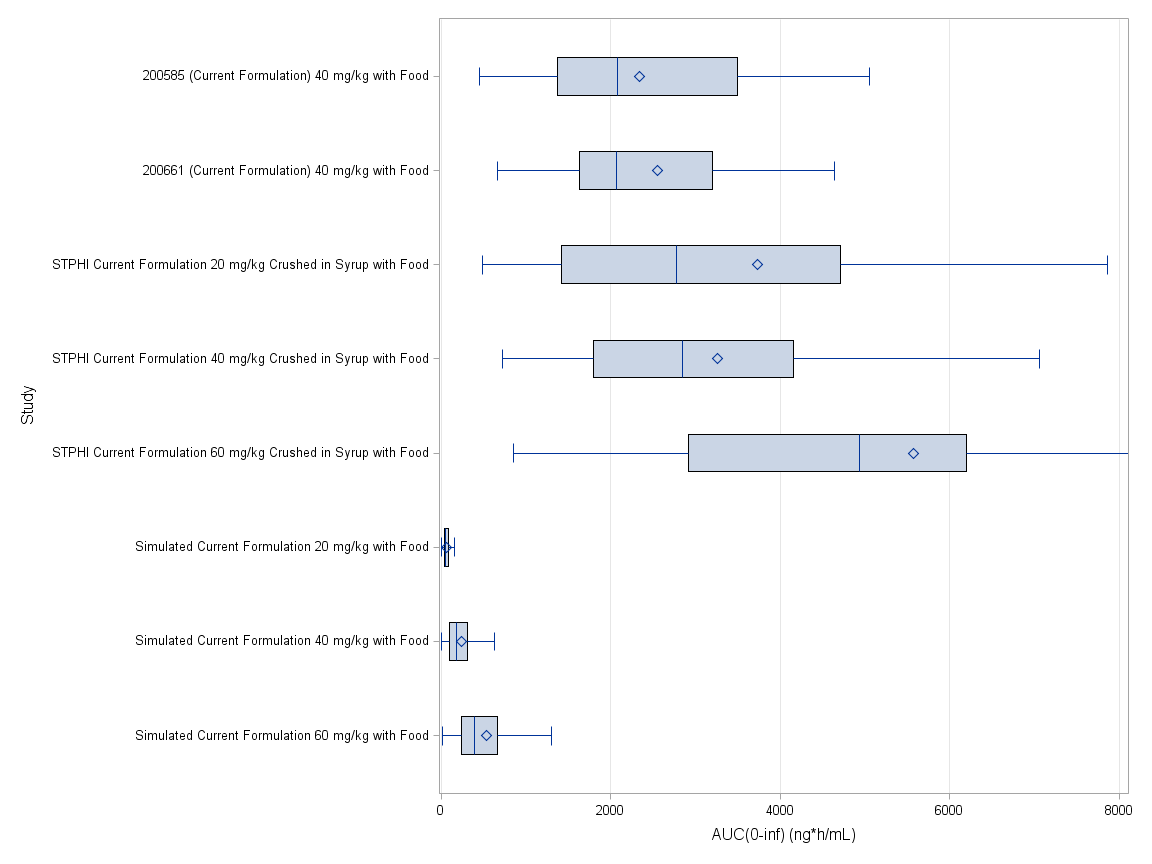 |
| --- |
| Comparison of observed L-PZQ AUC in Study 200585-001, Study 200661-001, and the Swiss Tropical and Public Health Institute Study to simulated L-PZQ AUC based on the combined data model. |
