# Supplementary figures and images for: Extrapolation of praziquantel pharmacokinetics to a pediatric population: a cautionary tale
Source: J Pharmacokinet Pharmacodyn. 2018 Sep 14;45(5):747–62. doi: 10.1007/s10928-018-9601-1 (PMC6182730; doi:10.1007/s10928-018-9601-1)

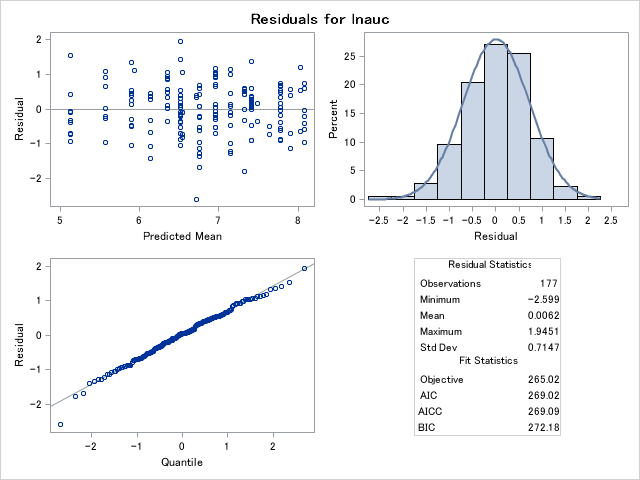

Supplement: Supplementary file 2 — Supplementary material 2 (PNG 33 kb) [file 10928_2018_9601_MOESM2_ESM.png]

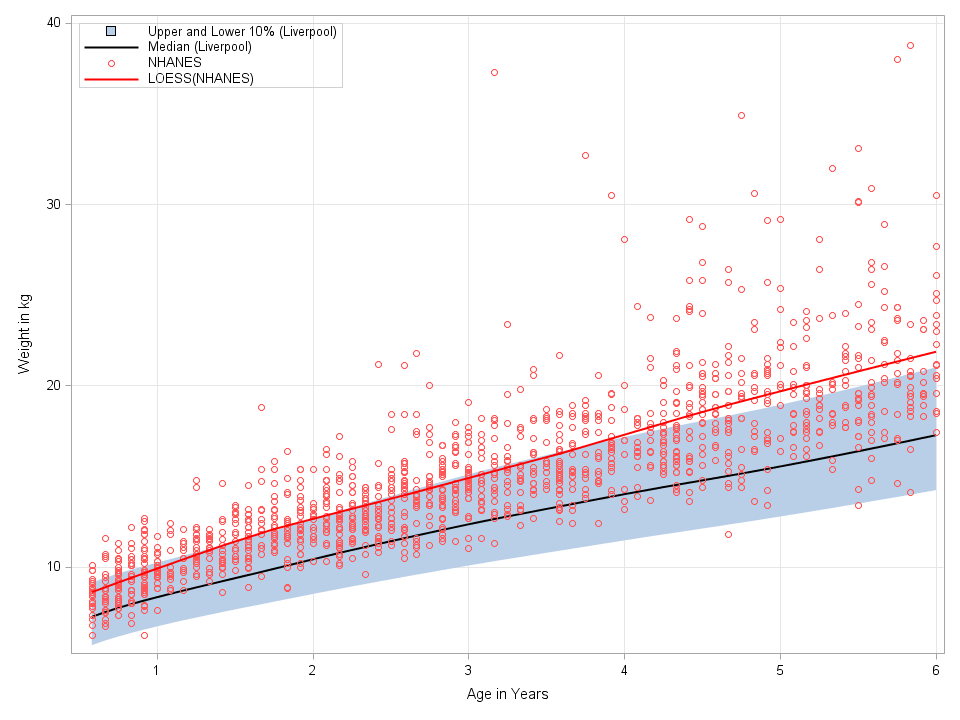

Supplement: Supplementary file 3 — Supplementary material 3 (PNG 72 kb) [file 10928_2018_9601_MOESM3_ESM.png]

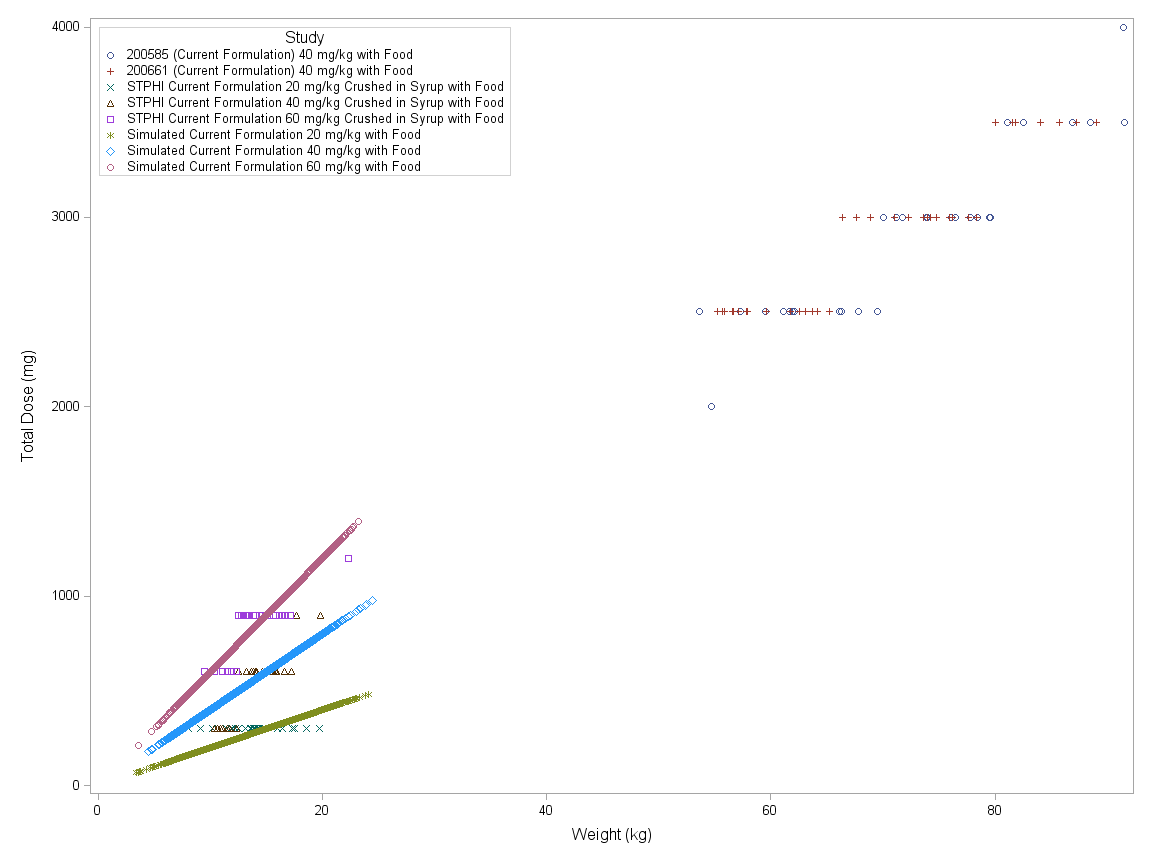

Supplement: Supplementary file 4 — Supplementary material 4 (PNG 30 kb) [file 10928_2018_9601_MOESM4_ESM.png]
